# A randomized, double-blind study of iodine supplementation during pregnancy in Sweden: pilot evaluation of maternal iodine status and thyroid function

**DOI:** 10.1007/s00394-021-02515-1

**Published:** 2021-02-23

**Authors:** Sofia Manousou, Robert Eggertsen, Lena Hulthén, Helena Filipsson Nyström

**Affiliations:** 1 Department of Internal Medicine and Clinical Nutrition, Institute of Medicine, Sahlgrenska Academy, University of Gothenburg, Göteborg, Sweden; 2 Frölunda Specialist Hospital, Marconigatan 31, 42144 Västra Frölunda, Sweden; 3 School of Public Health and Community Medicine/General Medicine, R and D Centre Gothenburg and Södra Bohuslän, University of Gothenburg, Göteborg, Sweden; 4 Department of Endocrinology, Sahlgrenska University Hospital, Göteborg, Sweden; 5 Wallenberg Center for Molecular and Translational Medicine, University of Gothenburg and Sahlgrenska University Hospital, Göteborg, Sweden

**Keywords:** Pregnancy, Urinary iodine concentration, Iodine supplementation, Thyroglobulin, Sweden, Randomized controlled trial

## Abstract

**Purpose:**

Pregnant women in Sweden are mildly iodine deficient. We investigated the effect of daily iodine supplementation on the iodine and thyroid status of pregnant women.

**Methods:**

In this pilot, randomized, double-blind trial, 200 thyroid-healthy pregnant women were recruited at mean (standard deviation) pregnancy week 8.85 (1.62) and assigned (1:1) to daily intake of a multivitamin tablet with or without 150 μg of iodine. Urine and serum samples were collected at baseline and once during the second and third trimesters. Urinary iodine concentration (UIC), serum thyroglobulin (Tg), thyroid-stimulating hormone (TSH), free thyroxine (FT4), and thyroid peroxidase antibodies (TPOabs) were analyzed. Neonatal TSH data were collected. UIC and Tg were also analyzed in a group of 89 thyroid-healthy non-pregnant women of reproductive age (WRA).

**Results:**

At baseline, the intervention and the control groups had similar median UIC (interquartile range (IQR)): 110 μg/L (74–119) and 111 μg/L (66–168), respectively. The intervention group reached iodine sufficiency with median UIC (IQR) 139 μg/L (89–234) and 136 μg/L (91–211) in the second and third trimester, respectively, without significant difference from the lower limit of the recommended range, i.e. 150–250 μg/L (*p* = 0.42 and *p* = 0.87, respectively). The intervention group had higher median UIC and lower median Tg compared to the control group during the second (*p* < 0.001 and *p* = 0.019, respectively) and third trimester (*p* < 0.001 and *p* = 0.003, respectively), whereas thyroid hormones, serum TPOabs, and neonatal TSH were similar. The WRA group presented median UIC (IQR) 65 μg/L (30–98) and median Tg (IQR) 18 μg/L (13–27).

**Conclusion:**

A daily supplement containing 150 μg of iodine to a group of pregnant women with mild iodine deficiency improved the iodine status from mild ID to iodine sufficiency. This improvement seems to have had a positive impact on maternal thyroglobulin. This study is now under extension to investigate the children’s neuropsychological development.

**Trial registration:**

ClinicalTrials.gov Identifier NCT02378246, May 3, 2015, retrospectively registered.

## Introduction

Iodine is needed for the production of thyroid hormones. Moderate-to-severe iodine deficiency in adults may lead to goiter and hypothyroidism. The mild form of iodine deficiency may lead to goiter with unaltered levels of thyroid hormones and elevated levels of thyroglobulin (Tg). Tg is the protein involved in the storage of thyroid hormone precursors within the thyroid gland, and reflects thyroid size in both iodine-deficient and iodine-excessive settings [1, 2]. During pregnancy, moderate-to-severe iodine deficiency increases the risk of cognitive impairment in the fetus [3–6], whereas the consequences of the mild form of iodine deficiency on offspring are unclear [7–13].

Sweden has historically suffered iodine deficiency with a prevalence of goiter up to 60% in some areas [14, 15]. In 1936, voluntary iodine fortification of table salt was introduced (10 mg/Kg salt) [14, 16] and, in 1966, the level of voluntary iodine fortification was increased to the current concentration of 50 mg/kg salt [17, 18]. The general population in Sweden is considered long-term iodine sufficient. This was confirmed in 2007 after our national study of school-aged children (6–12 years) showed no iodine deficiency-associated goiter [19] and a median urinary iodine concentration (UIC) of 125 µg/L [20], which is within the recommended range of 100–200 µg/L [21, 22].

However, iodine status in Sweden may have changed since 2007 due to altered dietary habits [23, 24]. Furthermore, iodine status among pregnant women differs from that of the general population. During pregnancy, the demand for iodine intake increases from 150 to 250 µg/day and the recommended UIC range is 150–249 µg/L [21]. A Swedish cross-sectional study in 2015 showed that pregnant women had mild iodine deficiency with normal thyroid hormone levels and that pregnant women not taking a daily iodine supplement of at least 150 µg presented higher Tg than those receiving such supplementation [25].

Despite mild iodine deficiency during pregnancy in Sweden, no recommendation on iodine supplementation can be safely given to pregnant women due to inconclusive data on its efficacy and safety [26]. Some observational studies have shown that mild iodine deficiency during fetal life is subsequently associated with poor childhood educational level and influence on verbal abilities and motor skills [7–10]. On the other hand, observational studies have also suggested iodine supplementation during pregnancy is associated with a higher risk of behavioral problems and poorer psychomotor/mental development in children [10, 27, 28]. Moreover, seven randomized controlled trials (RCTs) on iodine supplementation during pregnancy without child outcome were of low or moderate quality [29, 30] and the three published RCTs with child outcome [11–13] were unable to confirm a positive effect of iodine supplementation in mildly iodine-deficient pregnant women. However, it should be noted that the sample size was limited to 32 subjects in the intervention group in the trial from France [11], which studied daily intervention with iodine 200 µg started at gestational week 10. The PINK (Pregnancy Iodine and Neurodevelopment in Kids) trial in Australia [12], which studied daily intervention with iodine 150 µg starting at gestational week 15, had planned for a sample size of 1098 subjects but was stopped after 59 women were included (*n* = 27 in the intervention group) due to the introduction of a national recommendation for iodine supplementation in pregnancy, which conflicted with the study protocol. In the MITCH (Maternal Iodine Supplementation and Effects on Thyroid Function and Child Development) trial from India and Thailand [13], which tested daily intervention with iodine 200 µg starting at gestational week 10 (*n* = 416 in the intervention group), the placebo group developed unexpectedly iodine sufficiency during pregnancy. The need for RCTs on iodine supplementation in mildly iodine-deficient pregnant women, especially with child outcome, is internationally acknowledged [26]. The fear of adverse consequences from mild iodine deficiency during pregnancy has led to a general recommendation for the use of iodine supplements in some countries, while countries with a high level of iodine fortification, such as Sweden, need their own studies to support decisions [21].

It is important to confirm the efficacy of iodine supplementation in a pilot study before expanding to a larger sample size with follow-up among offspring. We planned a pilot RCT in Sweden from 2012 to 2015 with the objective of investigating the effect of iodine supplementation during pregnancy on iodine status and thyroid function in pregnant women. As the results from the national cross-sectional study in 2015 [25] were not available at that time, we hypothesized that pregnant women had mild iodine deficiency and that iodine supplementation with 150 μg daily could ensure iodine sufficiency, with a positive effect on maternal thyroid metabolism. In 2017, after the report from the MITCH study, we started the follow-up of these children and expanded the pregnancy recruitment to form the SWIDDICH study [31], that currently includes more than 800 pregnant women. This is the pilot pregnancy data from the first sub-sample of the SWIDDICH trial (ClinicalTrials.gov identifier: NCT02378246).

## Methods

### Study design

This was a pilot RCT on 200 pregnant women recruited at the Maternal Health Care Center in Skövde, an inland area of Southwestern Sweden, between November 2012 and September 2014. The women were randomized with a 1:1 allocation ratio to daily intake of a multivitamin tablet with iodine 150 μg (intervention group) or without iodine (control group) under double-blind conditions. Details of the randomization and the double-blind process are provided in the published study protocol [31]. Urine and serum samples were taken and a questionnaire completed at routine visits: baseline (pregnancy week 7–12) immediately prior to starting intervention, and then during the second (pregnancy week 25 ± 3 days) and third trimester (pregnancy week 36 ± 2 weeks). The urine samples were collected at random time points, because timed urine samples (meaning afternoon/ evening/ fasting or non-fasting in the morning) for the analysis of UIC are not considered better [32]. Data on age and pregnancy week were collected at each visit and the participants reported current smoking status or possible intake of iodine supplementation beyond the study tablet, the latter confirming non-adherence to the study protocol. UIC, urinary creatinine (U-crea), and serum Tg, Tg antibodies (Tgabs), thyroid-stimulating hormone (TSH), and free thyroxine (FT4) were analyzed at all visits, while serum thyroid peroxidase antibodies (TPOabs) were analyzed at baseline and during the third trimester. Neonatal TSH was archived from the neonatal screening program.

A group of 89 non-pregnant women of reproductive age (WRA group) was assembled at the research center of Skaraborg Hospital (Skövde, Sweden) from a random sample obtained from the Swedish Tax Agency of premenopausal women from Skövde between February 2013 and March 2016. Urine and serum samples were taken and a questionnaire completed on current smoking status and potential iodine supplementation intake. Their UIC and U-crea, and serum Tg and Tgabs were analyzed so we could compare the iodine status of non-pregnant women of reproductive age from the same area as the pregnant women included in the trial.

The study was approved by the Swedish Ethical Review Authority (Dnr 431–12). It was performed in accordance with the ethical standards of the 1964 Declaration of Helsinki and its later amendments. All study participants gave their informed consent prior to inclusion in the study. The full study protocol is published [31].

### Subjects

The inclusion criteria for pregnant women (*n* = 200) were: age 18–40 years, pregnancy week 7–12, and acceptance to refrain from iodine supplementation and to take the study supplementation instead. Exclusion criteria were: other pregnancy or lactation within 6 months prior to inclusion, veganism, and current thyroid disease. Vegans were excluded, due to ethical reasons; lacking both dairy products and fish in their diet, they presented a medical indication for iodine supplementation during pregnancy. We evaluated the presence of current thyroid disease based on self-reporting and, in cases of risk factors, on control of TSH according to local guidelines for screening of pregnant women at that time. These risk factors included the presence of other autoimmune disease, history of or heredity for thyroid disease, or symptoms of hypo- or hyperthyroidism.

The WRA group (*n* = 89) was matched for age and smoking habits with the first 90 pregnant women included in the study. The inclusion criteria for the WRA group were: woman 18–40 years of age and acceptance to not take iodine supplementation the week before inclusion. Exclusion criteria for the WRA group were: current or previous pregnancy or lactation within 6 months prior to inclusion, veganism, and current thyroid disease. The latter was based on both self-reporting and on control of TSH and FT4.

### Intervention

Women in the intervention group received a multivitamin containing 150 μg of iodine and those in the control group received a multivitamin without iodine. The multivitamin was taken daily from the day of inclusion (pregnancy week 7–12) until delivery. We did not use pure iodine tablets, because such were not commercially available. The content of the two multivitamins differed in several components besides iodine—see Table 1. It should be noted, however, that pregnant women in Sweden are routinely recommended a daily supplement of 400 μg folic acid during the first trimester [33] and that iron is recommended to the women who present low hemoglobulin, as part of a systematic hemoglobulin screening throughout pregnancy. Safety of supplements and randomization procedures are described elsewhere [31].

**Table 1:** Contents of multivitamin with iodine (intervention group) and multivitamin without iodine (control group) tablets

Quantity (percent of recommended daily intake during pregnancy) per tablet	
Multivitamin with iodine	Multivitamin without iodine
Iodine 150 μg (85%) Selenium 50 μg (71%) Calcium 250 mg (28%) Iron 12 mg (30%) Zinc 12 mg (133%) Vitamin B_2_ 1.4 mg (87%) Vitamin B_12_ 15 μg (750%)	Niacin 19 mg (111%) Folic acid 200 μg (50%) Vitamin A 400 μg (50%) Vitamin B_1_ 1.4 mg (93%) Vitamin B_2_ 1.7 mg (106%) Vitamin B_6_ 1.8 mg (128%) Vitamin B_12_ 3 μg (150%) Vitamin C 60 mg (70%) Vitamin D 5 μg (50%) Vitamin E 10 mg (100%)

### Urinary samples and estimation of urinary iodine excretion

Urine samples of ~ 10 mL were collected at each visit at the Maternal Health Care Center for the pregnant women and the research center of Skaraborg Hospital for the WRA group. The urine samples were transported and stored at − 20 °C until analysis as one batch. A single laboratory technician at the Department of Clinical Nutrition at Sahlgrenska Academy, University of Gothenburg (Göteborg, Sweden) performed all UIC analyses using the Pino modification of the Sandell-Kolthoff reaction [34]. The laboratory participates successfully (100% success the year the current samples were analyzed) in the EQUIP network (US Centers for Disease Control and Prevention, Atlanta, GA), which is the international standard for UIC measurements. All urine samples were measured in duplicate and reanalyzed in case of difference in absorbance > 5% for UIC > 150 μg/L, > 10% for UIC 50–150 μg/L, and > 15% for UIC < 50 μg/L. Control urine samples were added to the daily sample measurements as an internal quality control. The method for UIC was also validated at the Genomics and Biomarkers Unit in Helsinki, Finland, as part of the EU thyroid project, that has the aim to standardize iodine evaluation methods in Europe. The UIC method in the current study presented high correlation with the Euthyroid gold-standard (linear regression: *R*^2^ = 0.875, *p* = 0.023).

U-crea was measured by Department of Clinical Chemistry, Sahlgrenska University Hospital using a Cobas 6000/8000 analyzer (Roche Diagnostics, Solna, Sweden). Estimated urinary iodine excretion (eUIE) was calculated by the formula: eUIE (μg/day) = UIC µg/L/(U-crea g/L/1.23 g/day) based on the expected daily creatinine excretion for non-pregnant women 25–49 years of age (1.23 g/day) [35]. There is no constant for pregnant women and this is the most commonly used, also during pregnancy.

### Serum samples, definition of thyroid morbidity, and neonatal TSH

TSH for the WRA group was analyzed consecutively (as part of the exclusion criteria) at the Laboratory of Skaraborg Hospital. The rest of the serum samples were transported and stored at − 70 °C until analysis as one batch at the Laboratory of Clinical Chemistry at Sahlgrenska University Hospital. In case of abnormally high Tgabs (≥ 115 kIE/L, i.e. manufacturer’s cut-off), the Tg value was excluded from the analysis due to risk for analytical interference. The Tg assay was calibrated against CRM-457, i.e. the Certified Reference Material for human Tg. All variables were analyzed using a sandwich-type electrochemiluminescence immunoassay using Roche Cobas (Roche Diagnostics).

Kit- and laboratory-specific reference ranges were used for Tgabs < 115 kIE/L, TPOabs < 34 kIE/L, TSH 0.3–4.2 mIE/L (for the WRA group), and FT4 12–22 pmol/L (for the WRA group). TSH, as part of the inclusion criteria for pregnant women, was assessed based on the guidelines at that time, i.e. reference range 0.3–2.5 mIE/L for the first trimester. During the analysis of the results, however, the currently clinically used trimester-specific reference ranges were used: TSH 0.1–4.0, 0.2–4.0, and 0.3–4.0 mIE/L, and FT4 12–22, 10–17, and 8.6–16 pmol/L for the first, second, and third trimesters, respectively. Hypo- and hyperthyroidism were represented by abnormally high or low TSH, respectively, and this was classified as clinical or subclinical thyroid disease depending on whether FT4 was outside or within the reference range, respectively. Isolated hyper- or hypothyroxinemia represented high or low FT4, respectively, with normal TSH.

Data from the routine neonatal TSH screening was collected from all babies of the pregnant mothers. All newborn children in Sweden are tested, as soon as possible after 48 h following birth, for general screening of several conditions that need prompt care, including neonatal hypothyroidism. This is done by spot testing of heel prick blood collected onto filter paper cards (Perkin Elmer Ahlstrom 226 grade), which are dried at room temperature and transported on the same day to the Pediatric Phenylketonuria Laboratory at the Center for Congenital Metabolic Diseases at Karolinska University Hospital (Stockholm, Sweden). Neonatal TSH was analyzed by the 1235–5110 AutoDelfia automatic immunoassay system until January 2014 and thereafter by the 3301–0010 GSP (Genetic Screening Processor) fluorescence immunoassay system using a two-side fluoroimmunometric assay based on a direct sandwich technique. The laboratory defines TSH > 29 mIU/L as indicative of neonatal hypothyroidism for both analytical methods.

### Statistics

No power calculation was performed for this pilot study, due to lack of fully comparable RCTs on pregnant women at the time the current study was designed. We planned for a larger sample size (*n* = 200) than that of previous RCTs (*n* = 25–120 [36]). Power calculation was, however, conducted for the extended study (SWIDDICH study) with children’s mental outcome as primary endpoint, and is presented in the published study protocol [31]. Data analysis was performed using IBM SPSS statistics version 25 (IBM Corp., Armonk, NY). Normality was evaluated using histogram, Q-Q plots, and the Shapiro–Wilk test. Frequencies was analyzed by the Chi-square test or Fisher’s exact test in case of < 5 expected cases. Comparisons of independent medians or means were performed with the *t*-test for normally or Mann–Whitney *U* test for non-normally distributed variables. Related samples were analyzed with the Wilcoxon sign-rank test, as the variables were non-normally distributed variables. Comparison of non-normally distributed variables with a defined level was performed with one-sample Wilcoxon sign-rank test. All statistical significance was set as alpha significance 0.05.

## Results

Of all 200 women included, 158 remained in the study until delivery. The dropouts were due to miscarriage (*n* = 15), nausea (*n* = 4), experienced difficulty in remembering to take the study tablet (*n* = 4), general fatigue (*n* = 4), move to another area (*n* = 3), Crohn's disease relapse (*n* = 1), or unknown reason (*n* = 6). The dropout frequency and the frequency of miscarriage were similar between the two groups (*p* = 1.0 and *p* = 0.22, respectively). At all visits, all participants reported taking no other multivitamins than study tablets. Age, pregnancy week, and smoking status for each visit are presented in Table 2. The average pregnancy week at evaluation in the third trimester was half a week later for the control group compared to the intervention group. Otherwise, no statistical significant differences were revealed for background variables.

**Table 2:** Background characteristics of the study population

	Total	Intervention group	Control group	*p *value^a^
	At inclusion (*n* = 200)	At inclusion (*n* = 100)	At inclusion (*n* = 100)	
	Second trimester (*n* = 161)	Second trimester (*n* = 81)	Second trimester (*n* = 80)	
	Third trimester (*n* = 140)	Third trimester (*n* = 68)	Third trimester (*n* = 72)	
Age at inclusion, years	28.7 (4.2)	28.3 (4.0)	29.0 (4.2)	0.26
Pregnancy week				
At inclusion^b^	9.12 (1.18)	9.05 (1.17)	9.18 (1.21)	0.44
At inclusion^c^	8.85 (1.62)	8.87 (1.64)	8.82 (1.61)	0.84
Second trimester	25.28 (1.10)	25.25 (1.08)	25.31 (1.13)	0.71
Third trimester	35.96 (1.02)	35.69 (0.91)	36.21 (0.05)	**0.002**
Smoking				
At inclusion	9 (4.5%)	3 (3.0%)	6 (6.0%)	0.49
Second trimester	1 (0.6%)	0	1 (1.3%)	0.50
Third trimester	1 (0.7%)	0	1 (1.4%)	1.0

Data are given as mean (SD) for continuous or *n* (%) for categorical variables

Bold value indicate* p* < 0.05

^a^For between group comparison

^b^Calculation based on the last menstruation reported

^c^Calculation based on the ultrasound result in the second trimester, when available (*n* = 184)

**Fig. 1:**
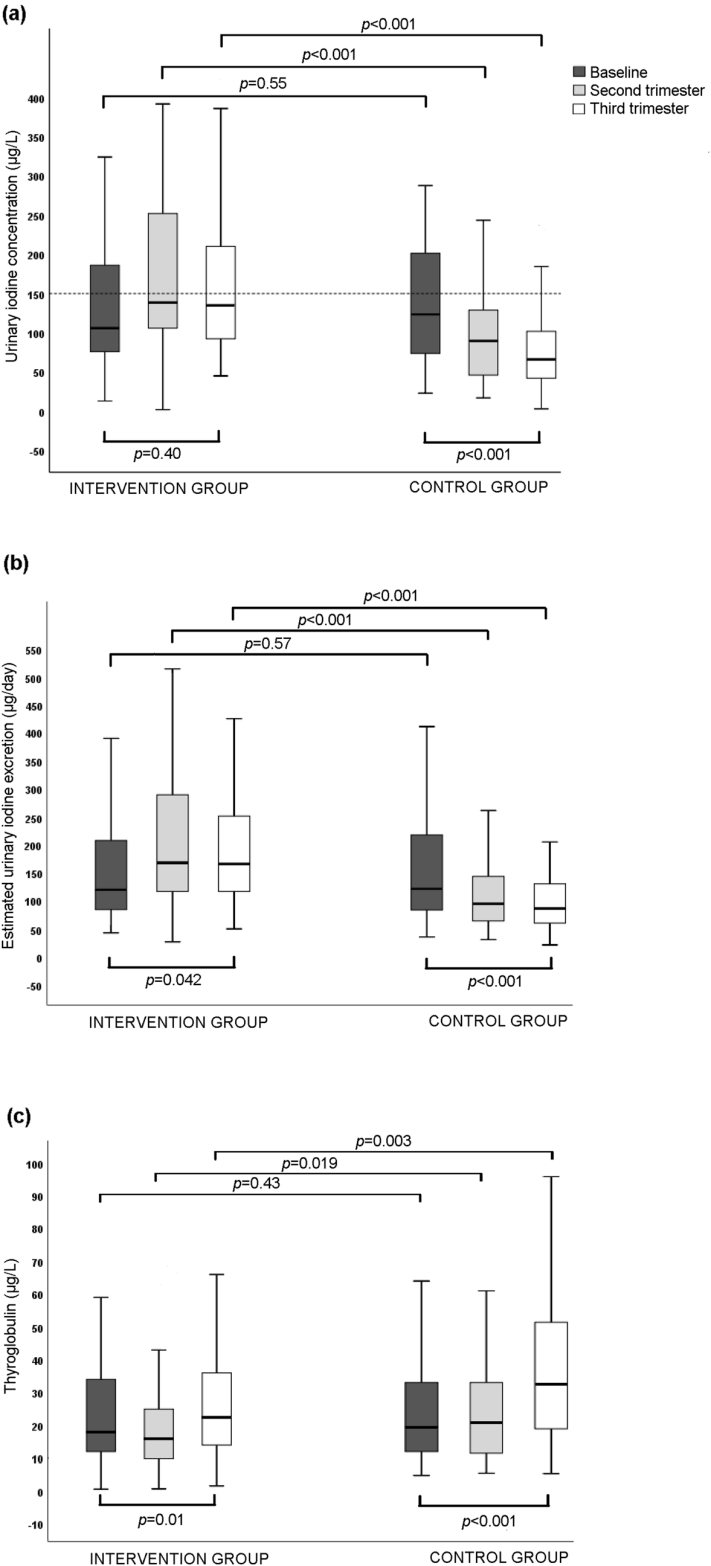
Boxplots comparing **a** urinary iodine concentration, **b** estimated urinary iodine excretion, and **c** serum thyroglobulin at baseline, and during the second and third trimesters for the intervention and control groups. Boxplots show median, interquartile range, and range. Dotted line in panel **a** represents the lower recommended median urinary iodine concentration during pregnancy

Median UIC was lower than the recommended limit of 150 μg/L [22] at baseline (*p* = 0.032) but not during the second (*p* = 0.42) and third (*p* = 0.87) trimester in the intervention group (Fig. 1a). By contrast, median UIC was significantly lower than the recommended limit at baseline (*p* = 0.002), and in the second (*p* < 0.001) and third (*p* < 0.0001) trimester in the control group.

Median UIC, eUIE, and Tg were analyzed in a cross-sectional manner at all visits and in a longitudinal manner between baseline and the third trimester (Table 3, Fig. 1a–c). At baseline, median UIC, eUIE, and Tg did not differ between the intervention and the control group. During the second and third trimester, the intervention group presented higher median UIC, and eUIE, and lower Tg compared to the control group. In the third trimester, the intervention group presented unaltered UIC, higher eUIE, and higher Tg compared to baseline, whereas the control group presented lower UIC, lower eUIE, and higher Tg compared to baseline.

**Table 3:** Urinary iodine concentration, urinary iodine excretion and thyroglobulin in the intervention and control groups

	Intervention group	Control group	*p *value^a^
UIC (μg/L)			
At inclusion	110 (74–189) [*n* = 97]	111 (66–168) [*n* = 96]	0.55
Second trimester	139 (89–231) [*n* = 81]	90 (44–132) [*n* = 79]	< 0.001
Third trimester	136 (91–211) [*n* = 65]	65 (39–108) [*n* = 70]	< 0.001
eUIE (μg/day)			
At inclusion	119 (76–191) [*n* = 97]	111 (71–197) [*n* = 96]	0.58
Second trimester	168 (115–272) [*n* = 81]	95 (63–159) [*n* = 79]	< 0.001
Third trimester	173 (117–252) [*n* = 65]	86 (60–134) [*n* = 70]	< 0.001
Tg (μg/L)			
First trimester	21 (12–34) [*n* = 93]	18 (12–32) [*n* = 86]	0.43
Second trimester	16 (9–25) [*n* = 78]	20 (13–32) [*n* = 74]	0.010
Third trimester	22 (14–36) [*n* = 66]	31 (19–51) [*n* = 68]	0.003

Data are given as median (interquartile range) with number of subjects assessed in square brackets

*UIC* urinary iodine concentration, *eUIE* urinary iodine excretion, *Tg* thyroglobulin

^a^For between group comparison

Cross-sectional analysis of maternal TSH, FT4, TPO-positivity, and neonatal TSH revealed no statistically significant differences (Table 4). The two analytical methods used for determination of neonatal TSH were equally distributed between the intervention and control groups (*p* = 1.0). No cases of neonatal hypothyroidism were observed in either group. The prevalence of thyroid morbidity (clinical and subclinical hypo- and hyperthyroidism as well as isolated hyper- and hypothyroxinemia) was similar between the two groups at all visits (data not shown).

**Table 4:** Maternal thyroid-stimulating hormone, free thyroxine, and thyroid peroxidase antibody positivity, and neonatal thyroid-stimulating hormone in the intervention and control groups

	Intervention group	Control group	*p* value^a^
TSH (mIE/L)			
At inclusion	1.2 (0.8–1.8) [*n* = 100]	1.2 (0.7–1.9) [*n* = 98]	0.56
Second trimester	1.7 (1.2–2.0) [*n* = 80]	1.6 (1.2–2.1) [*n* = 80]	0.94
Third trimester	1.9 (1.3–2.4) [*n* = 67]	2.1 (1.3–2.7) [*n* = 72]	0.52
FT4 (pmol/L)			
At inclusion	15 (14–17) [*n* = 100]	15 (14–16) [*n* = 98]	0.43
Second trimester	13 (12–13) [*n* = 80]	12 (11–13) [*n* = 80]	0.56
Third trimester	12 (11–13) [*n* = 67]	12 (11–13) [*n* = 72]	0.07
TPO positivity			
First trimester	12 (13.5%) [*n* = 89]	11 (12.7%) [*n* = 86]	0.83
Third trimester	5 (8.0%) [*n* = 62]	2 (2.9%) [*n* = 70]	0.26
Neonatal TSH (mIE/L)	4.0 (3.0–5.0) [*n* = 68]	3.0 (2.0–5.0) [*n* = 62]	0.30
Raised neonatal TSH	0 [*n* = 68]	0 [*n* = 62]	–

Data are given as median (interquartile range) or *n* (%) with number of subjects assessed in square brackets

*FT4* free thyroxine, *TPO* thyroid peroxidase, *TSH* thyroid-stimulating hormone

^a^For between group comparison

The WRA group presented median UIC (IQR) 65 μg/L (30–98), eUIE (IQR) 61 μg/day (38–100), and Tg (IQR) 18 μg/L (13–27). UIC was significantly (*p* < 0.001) lower than recommended range of 100–200 μg/L for non-pregnant women [21, 22].

## Discussion

This pilot RCT on iodine supplementation during pregnancy conducted in Sweden showed that daily supplementation with 150 μg of iodine improved the maternal iodine status from mild iodine deficiency to iodine sufficiency, even though UIC was at the lowest limit of the recommended by WHO range [21]. This improvement seems to have had a positive effect on maternal thyroid metabolism as reflected by serum Tg.

At baseline, both the intervention and control groups presented mild iodine deficiency; during the second and third trimester, only the control group remained with mild iodine deficiency, whereas the intervention group had reached iodine sufficiency with an UIC that did not differ (*p* = 0.42 and *p* = 0.47 respectively) from the recommended by WHO lowest limit of 150 μg/L [21]. The iodine status at baseline is in line with the results from the recent national cross-sectional trial where mild iodine deficiency was observed in pregnant women in Sweden [25]. In the intervention group, both UIC and eUIE were significantly higher than in the control group. Elevated iodine status after iodine treatment has also been observed in previous smaller RCTs [29, 30]. When compared to the three RCTs that reported child outcome [11–13], the results are similar. In both the French study [11] and the PINK trial [12], the study populations presented mild iodine deficiency at baseline and only the intervention group reached iodine sufficiency during the third trimester. Higher UIC in the intervention group compared to the placebo group was also observed in the MITCH trial [13], but it is important to note that both the intervention and the placebo groups in that trial ended up with iodine sufficiency during the second and third trimester, which will hopefully not be observed in our extended SWIDDICH trial given the results from the current pilot study.

The two groups in the current study had similar levels of Tg at baseline, whereas the intervention group presented lower Tg than the control group during the second and third trimester; thyroid hormone levels were, however, similar in the two groups, leading to the conclusion that the control group had mild and not moderate iodine deficiency. The interpretation of Tg results should be done with caution, as the intervention tablet also included selenium, which may interfere with thyroid metabolism [37]—the possible influence by selenium is planned for investigation in future analyses. Iron, included in the intervention tablet, is also a component that may act as confounder [38] and will be analyzed in the future. However, the risk of confounding by iron is considered limited, because the small amount of 12 mg of iron in the intervention tablet should be negligible, compared to the extra 100 mg of iron supplementation, that is commonly used by pregnant women in Sweden [39], and that should weaken or eliminate possible group differences regarding iron status. Neither neonatal TSH nor maternal TPO-positivity was different between the intervention and the control groups despite the known association between iodine and autoimmunity [40]. The results on maternal and neonatal TSH have been inconsistent among previous smaller RCTs on iodine supplementation in pregnancy, whereas the results on maternal Tg and TPO-positivity are mainly in line with the current study [29]. The Tg results in our study differ from the smaller French RCT [11], where the intervention group (*n* = 32) presented lower Tg than the control group, which was only just of marginal significance (*p* = 0.05) and only during the second trimester. This difference in Tg results may be due to the limited sample size of the French trial and to the imbalance in dropouts (20 in the intervention group *vs.* 4 in the control group). The PINK trial [12] did not investigate maternal Tg. The placebo group in the MITCH trial also presented slightly higher Tg (a difference of 1.10 μg/L) compared to the intervention group during the second and third trimester, which is of questionable clinical importance and needs to be interpreted in light of the observed iodine sufficiency in the control group.

Tg increased from baseline to the third trimester in both groups, but the difference in Tg between baseline and the third trimester was of clinical importance only in the control group (a difference of 10 μg/L); the difference of just 1 μg/L in the intervention group is unlikely to be of clinical importance. These results are in line with those from the previous, smaller RCTs [29, 30], where the increase of Tg during pregnancy has been generally of less magnitude in the group receiving iodine supplementation than in the control group. In the French RCT [11], Tg only decreased in the intervention group and only in the second trimester, whereas Tg in the control group remained unchanged during pregnancy. The intervention group in the current study, which experienced iodine sufficiency in the second and third trimester, presented Tg 16 and 22 μg/L, respectively, in line with an iodine-sufficient population from United Kingdom, that presented Tg 16 μg/L (analyzed using the same method as in the current study) [41]. The comparison of Tg between studies is challenging due to the large inter-method variability [42] and the influence from multiple factors [43]. Krejbjerg and colleagues [43] have suggested the inclusion of an iodine-sufficient reference population in studies where a reference Tg level is to be used. The ideal reference population for the current study would have been a group of pregnant women who would have taken iodine supplementation both before and during pregnancy. Although the issue is highly interesting, this was not the main purpose of the current pilot RCT. Nevertheless, high Tg in pregnant women in Sweden was observed in the recent national cross-sectional trial [25]—in both iodine-supplement users and non-supplement users—and speculation on the possible explanations was elaborated: pre-conceptional iodine deficiency and/or deficiency in other nutrients beyond iodine deficiency and/or generally larger thyroid gland in the Swedish population. The latter is in accordance with the observed larger thyroid glands of school-age children in Sweden compared to an international reference sample [19]. The lack of reference for the specific analytical method of Tg for pregnant women in the current study does not allow further conclusions to be drawn on the Tg level other than the conclusions based on the comparison between the study groups.

The positive effect of iodine supplementation on iodine status and maternal thyroid metabolism raises the question whether a national recommendation on iodine supplementation for pregnant women should be considered. The fear of adverse consequences from mild iodine deficiency during pregnancy has led to a general recommendation for the use of iodine supplements in some countries [44, 45], while countries with a high level of iodine fortification, such as Sweden, need their own studies to support decisions [21]. As elaborated elsewhere [25], we suggest that the current data on the clinical importance of mild iodine deficiency in pregnancy is inconclusive [7, 8, 13] and that the possible risks of iodine supplementation have not been ruled out [46]. Furthermore, the risks may be higher in case of pre-conceptional iodine deficiency, where a sudden increase in iodine intake may block the thyroid function of the fetus [46] and where the available iodine during pregnancy may be handled more effectively [47]. The pre-conceptional iodine status of our study population may be low as the WRA group (women of reproductive age from the same area not taking iodine supplementation) had a median UIC 60 μg/L [21, 22]. There is no data on the frequency of iodine supplementation pre-conceptionally in Sweden, but it is unlikely to be higher than the frequency of iodine supplementation in the first trimester of pregnancy (35–49%) [25]. Other data on UIC of women of reproductive age in Sweden derives from the national mapping conducted by the National Food Agency in 2010–2011, where 63 randomly selected women aged 18–44 years of age presented median UIC 74 μg/L [48]. This level of UIC suggests mild iodine deficiency according to WHO [21, 22], even though this definition is based on data from school-aged children and is questioned in adults due to its dependence on daily urinary volume. Based on these circumstances, the authors do not suggest iodine supplementation to pregnant women with mild iodine deficiency due to weak evidence on safety and efficacy, which is also supported by a recent review and meta-analysis [26]. The efficacy of iodine supplementation is going to be further tested in the extended version of the current study, the SWIDDICH study [31]. The positive effect of iodine supplementation with 150 μg daily on the surrogate outcomes (UIC, eUIE, and Tg) in this pilot study has led to the extension of the study to include 1275 women to follow-up the children’s neuropsychological development up to 14 years of age.

Even in the case that the extended SWIDDICH study suggests that restoration of iodine status of pregnant women is in favor of the children’s development, it may still be an issue whether iodine supplementation with 150 μg daily is the best choice compared to supplementation with higher doses or improvement of the iodine fortification program. In this pilot study, daily supplementation with iodine 150 μg resulted in borderline iodine sufficiency. It could be argued whether supplementation with iodine 200 μg daily would have been a more effective intervention. Daily supplementation with iodine 200 μg to mildly iodine-deficient pregnant women was tested in the MITCH trial [13] with ambiguous results, mainly due to the reason previously elaborated [49]. Another alternative to iodine supplementation is the improvement of the iodine fortification program in the country. Current salt iodization in Sweden is at the high level of 50 mg/kg salt [17] compared to the recommended 20–40 mg/kg [21], but is on a voluntary basis. Lower levels of voluntary iodization of salt (25 mg/kg salt) have been proven adequate in other countries to ensure at least borderline iodine sufficiency [50]. This suggests insufficient coverage of iodine fortification program in Sweden, which is also supported by recent data revealing that ~ 75% of consumed salt in Sweden is non-iodized (personal communication with the salt industry, 2017). We suggest that improving the coverage of the current iodization program should be the primary action needed if the SWIDDICH study shows that mild iodine deficiency during fetal life influences the neurocognitive development of the child.

We recognize the limited sample size of this pilot study, especially during the third trimester of pregnancy, even though it is higher than in most previous RCTs [29, 30]. In addition, dropouts were equal between the two groups and the reasons for discontinuation are unlikely to be associated with the contents of the study supplements or iodine/thyroid status. Dropouts are therefore not considered to induce selection bias and the weakened statistical power at evaluation during the third trimester has not prevented statistical differences from being revealed. The measured confounding factors were similar in the two groups during the second and third trimesters except for the median pregnancy week at evaluation in the third trimester: the control group was evaluated half a week later than in the intervention group. This difference of half a week is unlikely to have had any influence on the iodine status and is considered negligible. Another limitation of the study is the contents of the study supplements, which were not pure iodine or pure placebo tablets, as these are not commercially available. Considerations on the contents of the study supplements are thoroughly presented elsewhere [31] and are based on two factors: the possible future implementation of the results in practice imposes the use of available tablets and the components of the supplements that may be confounding factors are to be measured and taken into consideration in the statistical analysis of the extended study. If a reference pregnant population with known pre-conceptional iodine sufficiency was available, it would have facilitated the interpretation of Tg levels; Tg is though valuable in the current study when comparing the studied groups. The trimester-specific reference ranges for TSH and FT4 that we used, are the ranges currently used in clinical praxis in Sweden, and present weak evidence level; nation-specific reference ranges deriving from a thyroid healthy and iodine-sufficient population are currently lacking in Sweden. This methodological problem influences the evaluation of thyroid morbidity, but does not infer bias in the cross-sectional analysis of TSH and FT4 between the studied groups. The analytical method for neonatal TSH, as part of the national screening, was changed during the course of the study, but the risk for bias is considered low, as the two analytical methods were equally distributed between the intervention and control groups. In addition, neonatal TSH was also analyzed as a categorical variable, with neonatal hypothyroidism not present in either group. The important methodological strengths are the randomized, double-blind design of the study with the use of both cross-sectional and longitudinal analyses as well as the methods used for evaluation of the iodine status. All UIC samples were measured by the same experienced laboratory technician, with a well validated method. The availability of data on U-crea permitted the analysis of eUIE as a support to the main results. The main contribution of the current study compared with previous RCTs [29, 30], apart from the larger sample size, is that it is a pilot study to motivate inclusion of a much larger sample size with evaluation of iodine status, Tg, and thyroid hormones and with children follow-up in a population with mild fetal iodine deficiency in a country with a high level of salt iodization. The randomization code is now broken for these first 200 pregnant women, but all except for the central study team (i.e. study participants, lab engineers, psychologists) are still blinded.

In conclusion, daily supplementation with 150 μg of iodine to a group of pregnant women with mild iodine deficiency improved the maternal iodine status from mild iodine deficiency to iodine sufficiency in this pilot RCT. This improvement seems to have had a positive impact on maternal thyroglobulin, but did not influence maternal thyroid morbidity. As the intervention was found to influence surrogate outcomes positively, this study is now being extended in order to investigate the neuropsychological development of the offspring up to 14 years of age, thereby providing authorities and society with more data on whether improvement of iodine status must be undertaken in the case of mild iodine deficiency during pregnancy.

## Data Availability

Data cannot be made freely available as they are subject to secrecy in accordance with the Swedish Public Access to Information and Secrecy Act (Offentlighets—och sekretesslagen, OSL, 2009:400), but can be made available to researchers upon reasonable request subject to a review of secrecy. Requests for data should be made to corresponding author.
